# The single-cell expression profile of transposable elements and transcription factors in human early biparental and uniparental embryonic development

**DOI:** 10.3389/fcell.2022.1020490

**Published:** 2022-11-11

**Authors:** Conghui Li, Yue Zhang, Lizhi Leng, Xiaoguang Pan, Depeng Zhao, Xuemei Li, Jinrong Huang, Lars Bolund, Ge Lin, Yonglun Luo, Fengping Xu

**Affiliations:** ^1^ College of Life Sciences, University of Chinese Academy of Sciences, Beijing, China; ^2^ BGI-Shenzhen, Beishan Industrial Zone, Shenzhen, China; ^3^ Qingdao-Europe Advanced Institute for Life Sciences, BGI-Shenzhen, Qingdao, China; ^4^ Lars Bolund Institute of Regenerative Medicine, BGI-Qingdao, BGI-Shenzhen, Qingdao, China; ^5^ China National GeneBank, BGI-Shenzhen, Shenzhen, China; ^6^ Department of Biology, University of Copenhagen, Copenhagen, Denmark; ^7^ BGI-Qingdao, BGI-Shenzhen, Qingdao, Shandong, China; ^8^ Institute of Reproductive and Stem Cell Engineering, School of Basic Medical Science, Central South University, Changsha, China; ^9^ Key Laboratory of Reproductive and Stem Cells Engineering, Ministry of Health, Changsha, China; ^10^ Reproductive & Genetic Hospital of CITIC-Xiangya, Changsha, China; ^11^ Department of Reproductive Medicine, Affiliated Shenzhen Maternity and Child Healthcare Hospital, Southern Medical University, Shenzhen, China; ^12^ Department of Biomedicine, Aarhus University, Aarhus, Denmark; ^13^ Steno Diabetes Center Aarhus, Aarhus University Hospital, Aarhus, Denmark; ^14^ BGI Cell, BGI-Shenzhen, Shenzhen, China

**Keywords:** early embryonic development, transposable elements, transcriptional factors, androgenetic embryo, parthenogenetic embryo

## Abstract

Transposable elements (TEs) and transcription factors (TFs) are involved in the precise regulation of gene expression during the preimplantation stage. Activation of TEs is a key event for mammalian embryonic genome activation and preimplantation early embryonic development. TFs are involved in the regulation of drastic changes in gene expression patterns, but an inventory of the interplay between TEs and TFs during normal/abnormal human embryonic development is still lacking. Here we used single-cell RNA sequencing data generated from biparental and uniparental embryos to perform an integrative analysis of TE and TF expression. Our results showed that endogenous retroviruses (ERVs) are mainly expressed during the minor embryonic genome activation (EGA) process of early embryos, while Alu is gradually expressed in the middle and later stages. Some important ERVs (e.g., LTR5_Hs, MLT2A1) and Alu TEs are expressed at significantly lower levels in androgenic embryos. Integrative analysis revealed that the expression of the transcription factors *CTCF* and *POU5F1* is correlated with the differential expression of ERV TEs. Comparative coexpression network analysis further showed distinct expression levels of important TFs (e.g., *LEUTX* and *ZSCAN5A*) in dizygotic embryos vs. parthenogenetic and androgenic embryos. This systematic investigation of TE and TF expression in human early embryonic development by single-cell RNA sequencing provides valuable insights into mammalian embryonic development.

## Introduction

The transcriptional activity in mammalian early embryos leads to obvious dynamic changes in gene expression, particularly during the transition from zygote to morula (Niakan et al., 2012). The early stages of embryogenesis and precise activation of the zygotic genome are key to successful development (Tadros and Lipshitz, 2009; Lee et al., 2014).

Previous studies have shown that transposable elements (TEs) and transcription factors (TFs) are involved in the precise regulation of gene expression during the preimplantation stage (Godini and Fallahi, 2018; Rodriguez-Terrones and Torres-Padilla, 2018). TEs constitute more than 40% of the human genome (de Koning APJGu et al., 2011), and are divided into two types: DNA transposons transposed by cut-and-paste and retrotransposons mediated by RNA (Finnegan, 1989). Among all TEs, retrotransposons are predominant, including long terminal repeat (LTR) retrotransposons [including endogenous retroviruses (ERVs)] and non-LTR retrotransposons [long interspersed elements (LINEs) and short interspersed elements (SINEs)] (Wicker et al., 2007). The expression of retrotransposons is a vital event for genome reprogramming during early embryonic development (Bui et al., 2009). Some studies have also provided substantial evidence for the extensive role of TEs in regulating gene expression during early embryonic development (Kigami et al., 2003; Macfarlan et al., 2012; Percharde et al., 2018; Deng et al., 2019). Many TEs have been found, but the regulatory effects of most of these TEs have not been studied in depth.

In addition, the EGA process is directly regulated and orchestrated by different types of TFs, and many studies have provided important insights into the TFs present during early embryonic development (Lee et al., 2013; Leichsenring et al., 2013; Goolam et al., 2016; De Iaco et al., 2017; Hendrickson et al., 2017). Some important interplay between TEs and TFs is known to occur during early embryonic development. For instance, L1 RNA inhibits the expression of the transcription factor DUX by recruiting nuclear protein/Kap1, thereby indirectly inhibiting the transcription of ERVs (Percharde et al., 2018). Polak and Domany (2006) reported that Alu elements are present upstream of the transcription start site of a large number of genes, and the Alu sequence was found to contain multiple functional TF binding sites. These cross-sectional studies suggest an association between TEs and TFs. Therefore, it is necessary to systematically explore the cooperative regulation of TEs and TFs in early embryonic development at the transcriptional level.

Androgenetic (AG) and parthenogenetic (PG) embryos have two paternal or maternal genomes, respectively, and are valuable tools for studying the effect of parental genomes on preimplantation embryo development (Lagutina et al., 2004). In the initial stage after fertilization, zygotes undergo maternal-to-zygotic transition (Schier, 2007), during which maternal factors are gradually consumed and degraded, and the maternal and paternal genomes are reactivated to produce new mRNA and proteins to support embryonic development. A number of previous studies have shown that the contribution of the paternal and maternal genomes to the mammalian embryo genome is not exactly the same, and the diploid genome from only one of the two parental sexes cannot support complete embryogenesis (McGrath and Solter, 1984; Park et al., 2011; Hu et al., 2015).

To date, a systematic understanding of the role of TEs and TFs in early embryos is still lacking, particularly regarding how these two categories of regulatory elements orchestrate the reprogramming of maternal and paternal genomes during preimplantation development. In this study, we used single-cell RNA-seq data to conduct a comprehensive study of TE expression profiles in biparental and uniparental embryonic cells for the first time, in addition to identifying the key expressed TFs. The results showed some potential connections between TEs and TFs that may play an important role in embryonic genome activation and further differentiation.

## Result

### Transposable elements show developmental stage specific expression profiles in both uniparental and biparental embryos

**FIGURE 1:**
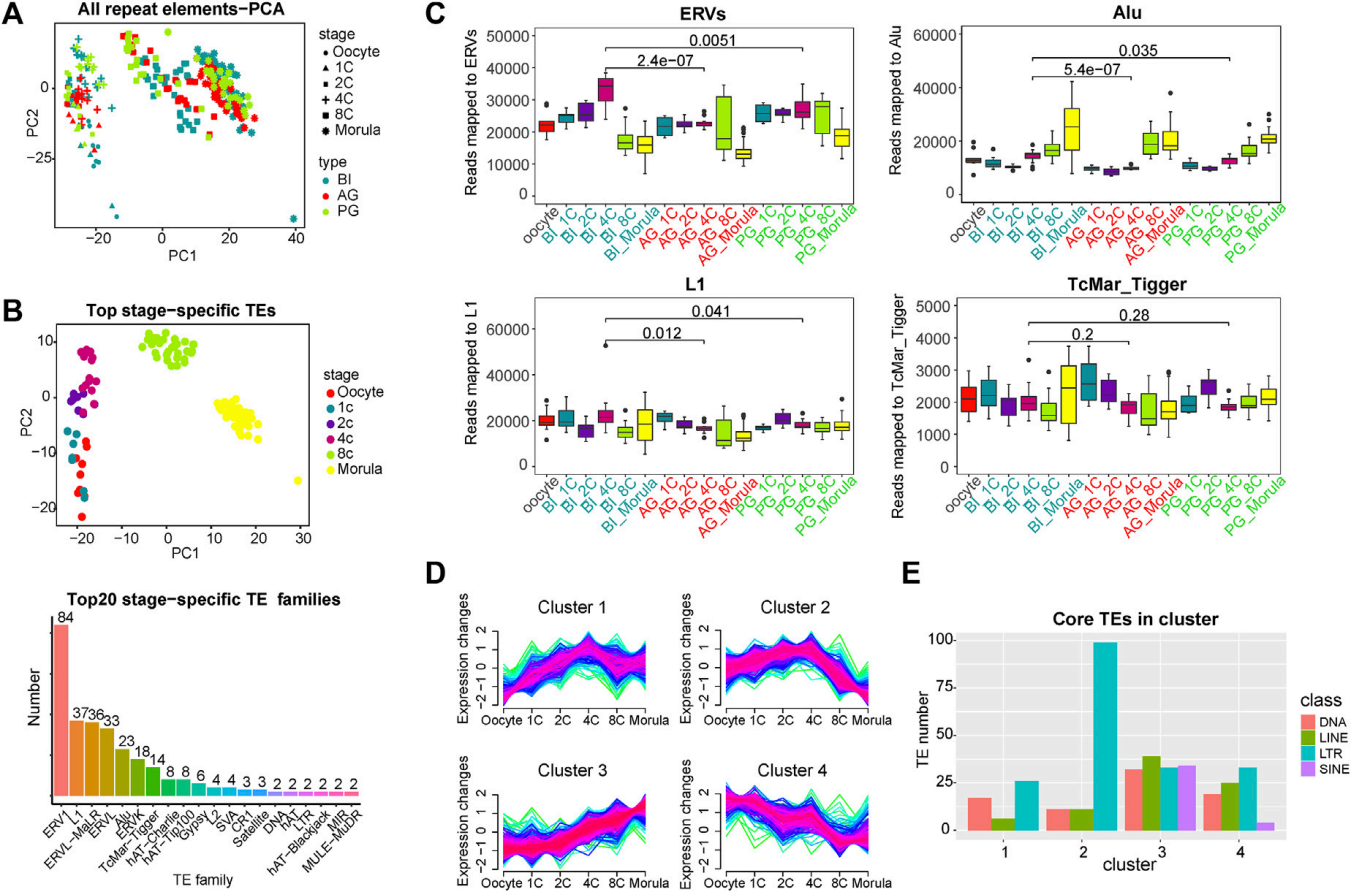
Global expression patterns of known TEs during the six consecutive stages of human preimplantation development. **(A)** Principal component analysis (PCA) of all TEs of single blastomeres of human preimplantation embryos. Blastomeres from the same stage of embryo are shown as symbols of the same shape. Blastomeres from the same type of embryo are shown as symbols of the same color. PCA1 and PCA2 represent the top two dimensions of the genes showing differential expression among these preimplantation blastomeres. **(B)** PCA on the stage-specific TEs expression estimates in biparental preimplantation embryos. The 309 TEs elements that showed the highest variation between the stages were selected, select the top 60 in each stage, some of which belong to two stages. The *p* values are from a paired Wilcoxon test, TEs which had a *p*-value<0.001 for the Wilcoxon test were selected as stage-specific (above). Number of each transposon family in stage-specific transposons (below). **(C)** Expression profiles of four transposon families in BI, AG, and PG embryos. ERVs include ERV1, ERVL, ERVL-MaLR, and ERVK. *Y* axis is the number of reads mapped to corresponding transposon families. Boxplots show the distribution for all single cells from the different developmental stages. Significance was calculated using the *t* test. **(D)** Fuzzy clustering analysis of TEs expression signals for the six consecutive stages. The closer the line color is to red, it means that these are the core TE in the cluster. **(E)**Number of core TE of each class in each cluster. Select transposons with membership value greater than 0.7 as core TE in ach cluster and count the number of four (membership≥0.7).

In our previous work, we reported that gene expression profiles showed phased differences during early embryonic development, and paternal and maternal genomes functioned differently during EGA and embryonic differentiation (Leng et al., 2019). This project provided us with a valuable single-cell RNA sequencing dataset to uncover the expression profiles of TEs in uniparental and biparental embryos. We quantified TE expression in all the samples, which contain transcriptome data of 285 single cells of the oocytes (*n* = 9), 1-cell (*n* = 15), 2-cell (*n* = 28), 4-cell (*n* = 37), 8-cell (*n* = 77), and morula (*n* = 119) stages in BI, AG, and PG embryos. To investigate whether the expression patterns of the TEs at each stage of the biparental and uniparental embryos were different, we performed a principal component analysis (PCA) based on the expression of 912 TEs. As shown in Figure 1A and Supplementary Table S1, the three types of embryos were clustered based on developmental stages rather than embryonic types, which showed that the expression of TEs was stage specific. Several lines of evidence suggest that the human EGA process accelerates from the 4-cell stage (Braude et al., 1988; Dobson et al., 2004; Vassena et al., 2011; Xue et al., 2013; Yan et al., 2013), so this result indicates that the 4-cell stage may be a critical period in early embryonic development.

To identify the TEs with stage-specific expression in early embryos, we calculated the differential expression of all TEs at each stage by the Wilcoxon test (*p* value < 0.05, Supplementary Table S2). Specific TEs at each stage (the top 60 with the lowest *p* value) were used for PCA. In Figure 1B/above, the different stages are better separated. We counted the family types of all these stage-specific TEs, and the top 7 TE families with a number greater than 10 were ERV1 (84), L1 (37), ERVL-MaLR (36), ERVL (33), Alu(23), ERVK(18), and TcMar-Tigger (14) (Figure 1B/below). The subsequent analysis of TEs was focused on the above families.

### Expression trend characteristics of transposable elements of different families

To more systematically analyse the transcription features of TEs from the four families at various stages of early embryonic development, a fraction of the transcriptome derived from these four families was calculated. In BI embryos, the ERV families were highly expressed from the 1- to 4-cell stage, and the expression decreased markedly from the 8-cell stage (Figure 1C). The expression level of the Alu family was low at the early stage and gradually increased from the 4-cell stage. The differences in expression in the L1 and TcMar_Tigger families in each stage were relatively mild. To further explore the expression characteristics of all the expressed TEs, we used a Mfuzz package to perform time-series analysis in BI embryos, and the TEs were divided into 4 clusters (Figure 1D). This showed that the expression trend of TEs was diversified in early embryonic development, and the expression trend of Cluster 1 and Cluster two showed a slight turning point at the 4-cell stage, suggesting that it may be related to EGA. Next, we investigated the class of core TEs in each cluster. The histogram in Figure 1E showed that LTR transposons (ERVs) had the highest percentage in Cluster 2, being upregulated from the 1- to 4-cell stage, maintaining a relatively high overall expression level, and downregulated from the 4-cell stage to morula stage. Previous studies by Xue and Yan have indicated that a minor EGA wave occurs during the 1- to 4-cell stage, and a major wave of EGA wave occurs at the 4-cell to morula stages (Xue et al., 2013; Yan et al., 2013). The expression trend of ERVs is highly consistent with the occurrence of minor EGA and major EGA, so we speculated that ERVs may be involved in the regulation of EGA. ERVs undergo a brief activation in the early stage of early embryonic development, which may contribute to the occurrence of minor EGA. Interestingly, SINE-class TEs seem to only appear in Cluster three and Cluster 4. The transcription of Cluster four shows a gradual decrease as the embryo develops, which mainly reflects the consumption and degradation of maternal materials, so the SINE-class TEs of the embryo themselves are gradually activated from the 4-cell stage in early embryonic development.

### Validation of transposable element expression in human and mouse embryos using public data

To validate the robustness of our findings, we analysed two independent single-cell RNA sequencing datasets (GSE36552 and GSE44183) of human embryos (Xue et al., 2013; Yan et al., 2013), both of which contained oocyte and single-cell samples at various developmental stages from zygote to morula. Indeed, using the same analysis strategy, we found that these two independent experimental datasets showed similar PCA results (Supplementary Figure S1A,B). The expression of TEs is also stage specific, and most importantly stage-specific TE families are also largely consistent.

We also took advantage of the scRNA-seq public data from zygote to morula stages in mice from Deng et al. (2014), and the average TE expression values in each stage were retrieved from Supplementary Table S3 of the paper published by Steven Xijin Ge (Ge, 2017). The results of the clustering analysis showed that the expression of TE in mouse embryos transitioned slightly at the 2-cell stage (Supplementary Figure S1C), which was earlier than the transition in human embryonic cells (4-cell stage). Evsikov et al. (2004) found that the LTRs sequences were abundant in the transcripts of 2-cell mouse embryos, and the major EGA in mice occurs during the 2-cell stage (Ram and Schultz, 1993). Various studies have fully confirmed that the EGA process in humans accelerates from the 4- to 8-cell stage, which is later than that in mice (Braude et al., 1988; Dobson et al., 2004; Vassena et al., 2011; Xue et al., 2013; Yan et al., 2013). The conservation of the expression trend of LTRs in humans and mice further indicates that ERVs may be related to the main EGA process in early embryos. In addition, these results also indicated that in both humans and mice, SINE-class TEs are gradually activated during early embryonic development, of course, Alu family which belongs to SINE-class is well known as a primate-specific family only considered in human here.

### Differences in transposable elements between biparental and uniparental embryos

EGA is one of the most important events that occur during preimplantation and embryo development in humans, and the embryonic genome should experience transcriptional quiescence to achieve large-scale transcriptional activity. To explore the activity of the TEs in the uniparental embryos during this process, we analysed the differentially expressed TEs during embryonic development, which was determined at a cutoff of FDR-corrected *p* value ≤ 0.01 and |log2 (fold change)|≥1.

**FIGURE 2:**
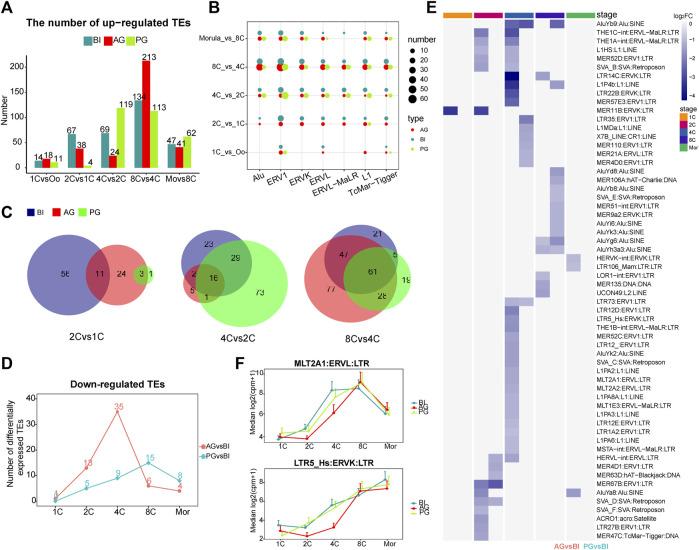
Analysis of differential transposon of BI, AG, and PG embryos. **(A)** Histogram shows the numbers of upregulated differential transposons across consecutive developmental stages. The blue, red and green colors represent BI, AG, and PG embryos, respectively. **(B)** The number of differential transposons belonging to the seven transposon families during the early development of the three types of embryos. The color of the bubble represents the type of embryo, and the size represents the number. **(C)** The venn diagram shows the number of common elements of differential transposons during early embryonic development of three types of embryos. The red, green and blue colors represent BI, AG, and PG differential transposons, respectively. **(D)** The line chart shows the number of significantly different downregulated transposons between uniparental embryos and biparental embryos at each stage (*p*. adj ≦ 0.05 and |log2 (fold-change)| ≧ 1). **(E)** Heat map shows all the significantly different transposons in **(D)**. Color represents the value of log2 (fold-change). **(F)** The expression levels of MLT2A1 and LTR5_Hs transposon in each developmental stage of three types of embryos.

We counted the number of upregulated TEs at each stage (Figure 2A). The statistical data of upregulated genes in the previous study (Supplementary Figure S2A) indicated that the expression dynamics of TEs are basically consistent with those of genes. Overall, the level of TE activation in uniparental embryos was lower than that in biparental embryos at the 1- to 2-cell stage. Compared to BI embryos, PG embryos showed compensatory upregulation at the 4-cell stage to a certain extent (69, 24, and 119 for BI, AG, and PG embryos, respectively), while this occurred at the 8-cell stage in AG embryos (134, 213, and 113 for BI, AG, and PG embryos, respectively), which may indicate that the TE activation in AG embryos in the minor EGA process is insufficient, and the TE activation in PG embryos is delayed. On the other hand, a large number of TEs are upregulated at the 8-cell stage indicating that TEs undergo a rapid and transient activation in the early embryonic development stage.

Next, we counted the number of the seven stage-specific TE families among the differentially expressed TEs at each stage. The dot plot in Figure 2B showed a huge distinction in the number of stage specific upregulated TEs in the three types of embryos from the 1- to 8-cell stage, with ERV1 having the largest difference, followed by Alu. Studies have shown that in embryonic stem cells, the ERV1 family contributes the greatest number of OCT4-and NANOG-binding sites (Kunarso et al., 2010). Then, we compared the intersecting upregulated TEs among the three types of embryos from the 1- to 8-cell stages. The AG embryos shared more TEs with BI embryos at the 1- to 2-cell and the 4- to 8-cell stages, with PG embryos at the 2- to 4-cell stages (Figure 2C). This characteristic may indicate that in the early embryo development process, the activation of TEs also makes a paternal or maternal biased contribution to the specific stage.

Comparison of the transcription of the 4 TE families in the three types of embryos, as apparent in Figure 1C, showed that the transcription trends of the ERVs were markedly different. The significant differences started at the 4-cell stage. The transcription level in AG/PG embryos was lower than that in BI embryos. There was a significant upregulation of ERVs at the 2- to 4-cell stages in BI embryos, but this tendency appeared in AG and PG embryos at the 8-cell stage, which can be as attributed to the upregulation of ERVs during the development of uniparental embryos later than that of normal embryos. In addition, compared with the BI embryos, the Alu family TEs of uniparental embryos were also downregulated to a certain extent at the 4-cell stage, and this downregulation was more pronounced in AG embryos. Moreover, according to our calculation, within the proximal promoter region (−2000 bp, 500 bp) of all upregulated genes, the most abundant type of TE was the Alu element (Supplementary Table S3). These findings, while preliminary, suggest that the lower expression of ERVs and Alu at the 4-cell stage may affect the minor EGA process in uniparental embryos, resulting in a higher probability of embryo EGA initiation failure.

Next, we compared the differentially expressed TEs between uniparental embryos and biparental embryos at each stage. At the 2- and 4-cell stages, more TEs were significantly downregulated in AG embryos (13, and 35 for 2-, and 4-cell embryos, respectively), especially at the 4-cell stage (Figure 2D). All the differentially expressed TEs are shown in the heatmap (Figure 2E). As expected, the most downregulated TEs in AG embryos belonged to the ERV families. In addition, we found that at the 4-cell stage, most of the TEs downregulated in AG embryos belonged to the upregulated TEs at the 2- to 4-cell stage of BI embryos (Supplementary Figure S2B), including LTR5_Hs MLT2A1, MLT2A2, LTR12D, L1HS, and AluYb9. Grow et al. (2015) reported that LTR5_Hs is bound by POU5F1, which is a master regulator of pluripotency, in the late stages of early embryonic development. The TEs MLT2A1 and MLT2A2 are specific to the 4- to 8-cell stages. The regulatory sequences contained in MLT2A1 and MLT2A2 are known to be potential targets of TFs such as DUX4, and OTX2 (Hendrickson et al., 2017; Liu et al., 2019), DUX4 activates HERVL by binding to the MLT2A1 element, which is a prominent event in ERV activation during EGA. The expression levels of LTR5_Hs and MLT2A1 were shown in Figure 2F. The downregulated expression of these two TEs in AG embryos may strongly affect the process of early embryo development.

In summary, the transcription of the ERV superfamily and the Alu family differed greatly at the 4-cell stage among the three types of embryos. The expression level in uniparental embryos was lower than that in BI embryos, and the difference between AG embryos and BI embryos was greater.

### The expression of key transcription factors at the 4-cell stage differs greatly among the three types of embryos

**FIGURE 3:**
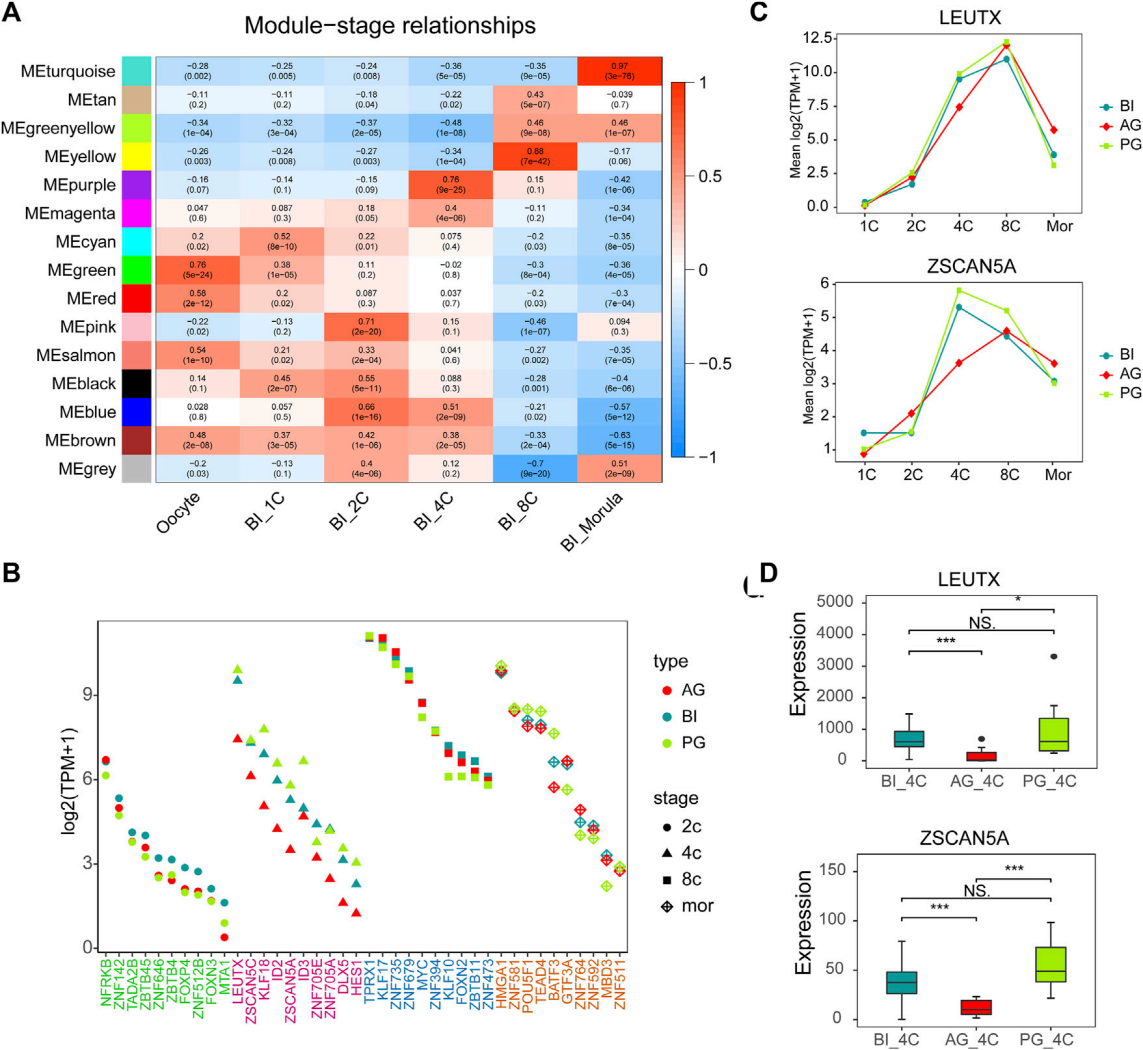
Gene co-expression network analysis in BI embryos and comparison of key TFs. **(A)** Heatmap showing relationships between modules and specific stages. Each cell contains the value of correlation and *p* value. **(B)** Module visualization of network connections between all TFs in the pink, purple, yellow, turquoise modules and other genes which show a high positive correlation (PC ≥ 0.7). Highly connected intramodular hub TFs (top10) are indicated by a different color dot. **(C)** LEUTX and ZSCAN5A expression in each stage of three types of embryos. **(D)** The expression of ZSCAN5A and LEUTX in the three types of embryos at the 4-cell stage.

To obtain the interaction pattern of genes throughout early embryonic development, we constructed a coexpression network of all the genes to observe their regulation and try to identify important TF genes. We performed weighted gene correlation network analysis (WGCNA) on 14,066 genes after filtering out genes with low expression levels in BI embryos, WGCNA is an unbiased and unsupervised analysis method that can identify different coexpression modules corresponding to correlated transcripts. Notably, 5 out of 15 modules showed high stage-specific expression (correlation ≥0.7), these modules contain genes that tend to be overexpressed in a single stage of development (Figure 3A). Among them, the pink, purple, yellow, and turquoise modules were highly correlated with the 2-cell to morula stages (Supplementary Figures S3A,B), and the correlation was highly significant based on the *p* value (*p <* 10^–20^). Then we selected all TFs in these four modules and other genes that showed a high positive correlation with these TFs (weight>0.1) to draw network diagrams (Supplementary Figure S4). According to the results of GO function enrichment analysis (Supplementary Figure S5), genes in the pink module were related to histone modification and methylation, genes in the purple modules were related to the regulation of gliogenesis differentiation, genes in the yellow modules were related to RNA splicing and chromatin assembly, and genes in the turquoise modules were related to protein transport in the endoplasmic reticulum.

The top 10 TFs with the highest connectivity in each module are shown in Table 1. We compared the expression levels of all 40 TFs among the three types of embryos (Figure 3B). Interesting, the differences at the 2-cell and 4-cell stages were relatively regular. The expression of these TFs in AG and PG embryos was slightly lower than that in BI embryos at the 2-cell stage. All the 10 TF genes were greatly downregulated in AG embryos and eight transcription factors were upregulated in PG embryos compared with BI embryos. This suggests that the genomes of the two parental embryos have opposite effects on minor EGA at the 4-cell stage. Importantly, the number of significantly upregulated genes at the 1–8 cell stages (Supplementary Figure S2A) was positively correlated with the expression levels of TFs in the corresponding period, indicating that the degree of minor EGA is regulated by these key TFs. The difference in TF expression may contribute to insufficient reprogramming and premature differentiation of AG embryos. Further analysis of the expression characteristics and regulatory network of the key transcription factors ZSCAN5A and LEUTX, which have been examined in related reports, revealed that the expression of ZSCAN5A and LEUTX in AG embryos was significantly lower than that in PG and BI embryos at the 4-cell stage (Figures 3C,D). We also checked the expression of related genes coexpressed with ZSCAN5A at the 4-cell stage of the three types of embryos in the coexpression network and found that the expression of the coexpressed genes was consistent with ZSCAN5A (Supplementary Figure S6). This further illustrates the importance of key TFs at the 4-cell stage for the transcriptional activity of the minor EGA process.

**TABLE 1:** Transcription factor with the highest connectivity within the significant module related to the stage.

Rank	Pink (2)	Purple (4)	Yellow (8)	Turquoise (morula)
1	FOXP4	ZSCAN5C	ZNF394	POU5F1
2	NFRKB	KLF18	ZNF473	ZNF581
3	TADA2B	DLX5	KLF17	GTF3A
4	ZNF646	ZNF705A	ZNF679	HMGA1
5	ZNF142	ZSCAN5A	TPRX1	BATF3
6	MTA1	ID3	MYC	ZNF592
7	ZBTB4	HES1	ZNF735	ZNF511
8	FOXN3	ID2	FOXN2	TEAD4
9	ZNF512B	ZNF705E	KLF10	ZNF764
10	ZBTB45	LEUTX	ZBTB11	MBD3

### POU5F1 and CTCF may affect the development of uniparental embryos through differentially expressed transposable elements

**FIGURE 4:**
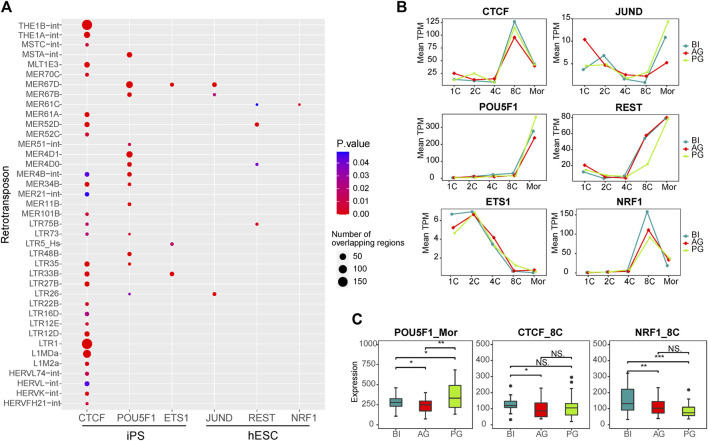
Transcription factors associated with differential transposons. **(A)** Dot plot showing the correlation between transcription factors which expressed in our data and differential transposons between three types of embryos. The dot size represents the total number of TE that intersect with the binding sites of TF in the ChIP-seq experiment. **(B)** Line chart showing the expression of six transcription factors in each stage of three types of embryos. **(C)** The box plot shows the significant difference in the expression of POU5F1, CTCF, and NRF1 in the three embryos at the corresponding stage. Significance was calculated using the *t* test.

Retrotransposons can act as enhancers to provide genes with cis-regulatory sequences. They influence the transcriptional activity of nearby genes by providing binding sites for TFs. To further explore the correlation between TEs and TFs in early embryonic development, according to a report by Karakülah (2018), we downloaded data regarding the correlation between TFs and TEs in iPS cells and hESCs from the RTFAdb website. Among them, the CTCF, POU5F1, and ETS1 transcription factors in iPS cells and JUND, REST, and NRF1 transcription factors in hESCs cells were not expressed at low levels in early embryonic cells. Therefore, we focused on the correlation between these six TFs and the differentially expressed TEs between the three types of embryos (Figure 4A; Supplementary Table S4). CTCF was associated with the most TEs, followed by POU5F1 (OCT4). Among them, the binding site of CTCF showed more overlap with LTR1, THE1B-int, L1MDa, THE1A-int, and MLT1E3 (180, 175, 90, 42, and 30, respectively). The binding site of POU5F1 showed 56 intersections with MER67D and 44 intersections with MER4D1, and these TEs included almost all of the ERV family.

Our WGCNA showed that POU5F1 is a key TF in the coexpression network in the morula stage, and CTCF and NRF1 are key TFs in the 8-cell stage coexpression network (Supplementary Figure S4). ChIP-Seq data from a previous study showed that TEs account for 25% of the key TF binding sites of human embryonic stem cells, such as *CTCF* and *POU5F1* (Kunarso et al., 2010), POU5F1 is a known key regulator of embryonic stem cells and is related to pluripotency (Medvedev et al., 2008; Niakan and Eggan, 2013). CTCF is an important factor in the regulatory network (Hee Jung et al., 2019), which regulates chromatin structure in a variety of ways, including by isolating epigenetic transmission and participating in chromatin ring formation (Satou et al., 2015). The binding sites of CTCF are enriched in ERV elements (Ito et al., 2017), and their combination facilitates the organization of higher-order chromatin structures, thereby promoting the overall reconstruction of chromatin structure during preimplantation embryo development (Schmidt et al., 2012; Ke et al., 2017). Data from several sources have identified that NRF1 is related to early embryonic development, and defects in NRF1 are related to the decreased expression of various genes encoding centromeres and affecting mitosis, and may play a role in maintaining genome integrity (Oh et al., 2012; Williams et al., 2013; Zhang and Xiang, 2016; Sant et al., 2017). Therefore, we investigated their expression in the three embryos, POU5F1 was different at the morula embryonic stage, while CTCF and NRF1 were different at the 8-cell stage (Figures 4B,C).

Taken together, the results suggest that the transcription factors POU5F1, and CTCF cooperate with the differentially expressed TEs of the ERV families such as LTR1, THE1B-int, THE1A-int, MLT1E3, and MER67D, as shown in Figure 4A, to regulate the major EGA process of early embryos. Their differential expression may affect the normal development of the three types of embryos.

## Discussion

Through the analysis of the single-cell transcriptome at each developmental stage of the early embryos, our research showed that TEs are transcribed in a stage-specific manner during the early development of BI, AG, and PG embryos. Among them, the expression of the ERV and the L1 family exhibit highly stage specific. ERVs are one of the most abundant transposable elements, accounting for 8% and 10% of the mouse and human genomes, respectively (Lander et al., 2001; Mouse Genome Sequencing Consortium Waterston et al., 2002). The expression of ERVs contributes to cellular plasticity and the activation of the embryonic genome, which is related to the establishment of pluripotency and totipotency (Lu et al., 2014). Studies have shown that knockout of L1 can lead to the failure of EGA and the maintenance of a 2-cell state in mouse preimplantation embryos (Percharde et al., 2018). In addition, other studies have shown that the activation of L1 after fertilization regulates the accessibility of chromatin in early mouse embryos (Jachowicz et al., 2017). Therefore, ERVs and L1 play important roles in the EGA process, and their expression may be a hallmark of cellular identity and cell potency that characterize the cell state in early human embryos.

The difference in TE expression among the three types of embryos was mainly reflected at the 4-cell stage. According to our data, ERVs are gradually expressed in the early stages of normal biparental embryos and have higher expression in 1- to 4- cell stage embryos, which suggests that the expression of ERVs is related to the minor EGA process. However, the transcription level of the TEs of uniparental embryos is lower than that of biparental embryos, especially AG embryos, such as LTR5_Hs, MLT2A1, and other known important TEs of ERVs and the Alu family. It is known that most of the empty LTR elements in the human genome still maintain transcription and regulatory functions, affecting the expression of neighbouring genes (Rebollo et al., 2012). In mouse preimplantation embryos, ERVs account for a large part of the transcriptome of the minor EGA, and many important minor EGA-specific genes are regulated by LTR elements in ERVs (Peaston et al., 2004). The activation of MERVL in mice results in extremely high transcription as early as 8 h after fertilization (minor EGA) (Kigami et al., 2003). Downregulation of MERVL can cause developmental arrest at the 2-cell stage (Huang et al., 2017). Although the transcription profiles of ERVs differ among species, the transcriptional activation of ERVs is a conserved event in early mammalian embryos (Rowe and Trono, 2011). Based on this, we speculated that the low expression of ERVs may affect the activation of the zygotic genome of uniparental embryos and the totipotency of embryo development.

According to our results, the expression of SINEs gradually increases with developmental stage. Ge reported that SINEs are associated with large-scale genome activation in early embryonic development in mice and humans, and the degree of activation is related to the position distribution and dosage of the SINE elements in the gene promoter (Ge, 2017). It has previously been observed that the expression and accessibility of the SINE families increase starting at the 8-cell stage in bovines, suggesting that these elements may act as promoters or enhancers (Halstead et al., 2020). As one of the most important and abundant SINE TEs that are still active in the human genome, Alu showed an indispensable regulatory function at the sequence level. Alu shares high sequence identity with the binding motifs of many important TFs and was proven to be bound by key TFs such as LEUTX, PITX2, and OTX2 (Polak and Domany, 2006; Töhönen et al., 2015; Jouhilahti et al., 2016). In addition, studies have shown that the Alu element is also an important CpG site provider. The GC-rich Alu sequence can introduce high GC content and methylation flexibility into the remote chromatin contact area, and regulate tissue-specific genes (Gu et al., 2016), showing its spatial role. Therefore, the changes in the expression of Alu and key TFs such as LEUTX, affect the normal development of early embryos.

The expression of key TFs such as ZSCAN5A and LEUTX at the 4-cell stage was lower in AG embryos, Sun et al. (2016) proved that knocking down ZSCAN5A can cause abnormalities in spindle assembly or attachment during mitosis, which can cause metaphase arrest and aneuploidy, leading to abnormal cell division (Sun et al., 2016). Based on the cleavage conditions of the three types of embryos, in the normally dividing embryos, the second cell division cycle of AG embryos is shorter and PG embryos are longer than BI embryos, showing the opposite trend (Leng et al., 2019). An implication of this is the possibility that the downregulation of ZSCAN5A at the 4-cell stage leads to a shorter second cleavage cycle in AG embryos, which leads to insufficient material reserves in the minor EGA process. In addition, studies have shown that LEUTX may be the main regulator of EGA, as 25% of the genes upregulated in 8-cell embryos were experimentally verified as LEUTX target genes. The expression of LEUTX at the 4-cell stage is critical to early embryonic development (Jouhilahti et al., 2016). Another study reported that the *de novo* motif overlapping with the Alu elements is similar to known consensus sequences of binding sites for PRD-like homeodomain containing TFs (Töhönen et al., 2015), such as LEUTX. Meanwhile, the expression level of Alu family TEs in AG and PG embryos was lower than that in BI embryos at the 4-cell stage, especially in AG embryos. Therefore, we speculate that the downregulation of LEUTX and Alu retrotransposon elements in AG embryos plays an important role in transcriptional regulation. Further experiments and multiomics analysis are needed to prove this hypothesis. The molecular profiles of PG embryos, including the TF and TE profiles, are more similar to those of BI embryos, indicating that the maternal genome plays a more important role in regulating transcriptional activity during EGA. The longer duration of PG embryonic development than that of BI embryonic development may be caused by the lack of sperm in PG embryos, and the formation of mitotic centres may take longer (Bui et al., 2011; Escribá et al., 2016; Leng et al., 2019).

According to our statistics, the distribution of TEs in the promoter regions of genes in the regulatory networks at different stages has no obvious bias, indicating that the distribution of TEs may regulate the activation of the genome during the development of early embryos with epigenetic modifications such as methylation and chromatin accessibility. To further determine the relationship between the expression of a TE at a specific location and the expression of adjacent genes, the epigenetic regulation and chromatin state of the specific elements need to be determined. In addition, the current experimental sequencing methods used in TE research usually produce shorter reads (<150 bp). Given the highly repetitive sequence characteristics of TEs, it is necessary to align the sequences to specific positions with high reliability. In terms of experimental methods, we need longer sequencing read lengths, which can not only reduce the difficulty of alignment but also improve accuracy. In summary, the use of long-read sequencing methods, combined with the appearance of the chromatin open state and methylation, can provide a better understanding of the relationship between the expression of TE at a specific location and the expression of adjacent genes.

In summary, our systematic studies of the transcription trend and dynamic changes of TEs during early embryonic development revealed the influence of different TEs on the EGA process and provided a comprehensive regulatory framework for human early embryos. The differences in the expression of TEs and TFs among the three embryos were compared, and some important factors that may cause early embryo abnormal development were found, which will be helpful for analysing the molecular mechanism of early embryo development and may have an impact on developmental biology. It may also provide directions and ideas for follow-up research to improve the success rate of *in vitro* fertilization.

## Methods

### Sample information and data collection

To study the expression of TEs and TFs in the human early embryo we took advantage of downloaded single-cell RNA-seq public data from Gene Expression Omnibus (GEO) with accession number GSE133856 using the fastq-dump program of SRAtools suite, which was generated by a previous project in our laboratory. This dataset contains 296 single cells from oocytes and embryos of the 1-,2-,4-,8-, morula-cell stage in BI, AG, and PG embryos. BI embryos acquired by Intracytoplasmic sperm injection (ICSI), AG embryos acquired by inducing two sperm into an enucleated oocyte, and PG embryos acquired by inhibiting second polar body extrusion. Embryos were cultured at 37.5°C in 6% CO_2_, 5% O_2_, and 89% N_2_. The culture medium was changed on day 3. To isolate individual embryo cells, the embryos were exposed to acidic Tyrode’s solution for 3–5 s and then washed thoroughly in phosphate-buffered saline (PBS) containing 0.5% bovine serum albumin (BSA) to remove the zona pellucida. Zona-free embryos were incubated for 10 min (for the 2-, 4-, and 8-cell stages) or 15 min (for the morula stage) in Accutase medium, and then disaggregated by careful pipetting. Single blastomeres were placed into individual tubes containing 4 µl of lysis buffer or 0.5 µl of PBS for immediate preparation for RNA or DNA libraries, respectively. RNA-seq libraries were prepared using the SMARTSeq2 protocol, single-cell libraries were constructed using a Nextera XT DNA Library Preparation Kit (Illumina, Cat#FC-131-1096), and all libraries were sequenced on an Illumina Hiseq2500 or Hiseq X Ten instrument, according to the manufacturer’s instructions (Leng et al., 2019).

### Data pre-processing

The initial quality check was carried out using FastQC (FastQC, 2020). Trimmomatic was used for trimming of sequences (Bolger et al., 2014), Human genome sequence (GRCh38) and annotation were downloaded from ENSEMBL, We used the STAR (Dobin et al., 2013)and HTSeq (Anders et al., 2015) programs to map and quantify gene expression. Because repetitive elements are distributed across many chromosome positions, to estimate the expression of TEs, we re-mapped reads using STAR to allow more multiple mapped reads using the following parameters: STAR–outFilterMultimapNmax 100 –winAnchorMultimapNmax 100 –outSAMmultNmax 100 –outSAMtype BAM SortedByCoordinate–outFilterMismatchNmax 3 (Ge, 2017). We used TEtranscripts (Jin et al., 2015), specifically designed to estimate both genes and TEs abundance, to calculate the expression level of repeated sequences by using an additional index of TEs based on UCSC repeatMasker files. The parameters we used are: TEtranscripts–format BAM–SortedByCoordinate -outFilterMismatchNmax 3mode multi–GTF genes. gtf–TE GRCh38_rmsk_TE.gtf -i 10 –stranded no. Because Repetitive elements have multiple duplicates of varying lengths, our general strategy was not to use length correction in RNA-seq. The raw counts of TE were normalized on the total number of mapped reads and multiplied by 1,000,000 obtaining expression values indicated as counts per million (CPM) and the counts of gene were normalized into transcripts per million (TPM).

Here we collected 285 cells in total, including nine oocyte cells, 114 BI cells, 89 AG cells, and 73 PG cells (Supplementary Table S5). To select TEs and genes with a reproducible expression among the replicates of the same cell type, we selected TE and mRNAs with an expression value ≥1 (CPM or TPM) in at least 70% of replicates of at least 1 cell type. Finally, we identified 912 expressed TEs and 14,066 expressed genes for subsequent analysis. We also retrieved TE expression values in mice from the Supplementary Table S3 of the paper published by Steven Xijin Ge (Ge, 2017) and collected two other human datasets GSE36552 and GSE44183 (the scRNA-seq data published by Yan et al. (2013) and Xue et al. (2013), respectively) for verifying our conclusion (Supplementary Table S5).

### Stage specific analysis

First, Principal component analysis (PCA) was performed to cluster biparental and uniparental embryos using all expressed TEs. The Wilcoxon test is a non-parametric statistical test used to compare two paired groups. The goal of the test is to determine whether two or more pairs are statistically different. Stage-specificity of individual elements was estimated using the Wilcoxon test on the CPM normalized data, every developmental stage was tested against all other stages. TEs with *p* value < 0.001 by the Wilcoxon test were selected as stage-specific. The PCA plot was generated using R on the top 60 TEs that showed the strongest variance between the different developmental stages, with a total of 309 elements.

### Fuzzy clustering analysis and differential analysis of transposable element

We calculated the mean value of the expression of TEs for the biological replicates then made a log transformation, which was used as an input for Fuzzy analysis. The R package Mfuzz (Kumar and Mfuzz, 2007) was used for clustering analysis. Before clustering, we removed TEs with normalized values < 0 at all stages. Finally, we divided all 912 TEs into 4 clusters and defined the core elements of a cluster with a membership value greater than 0.7. DESeq2 (Love et al., 2014) package was used to detect differential expression TEs in six consecutive developmental stages from oocyte to morula.

### Gene co-expression network analysis

All 14,066 filtered genes in BI embryo samples were applied to construct the co-expression network using the WGCNA package (Langfelder and Horvath, 2008), and then we selected TFs and their mainly related genes in the modules whose correlation with the stages is greater than 0.7 for visualization. All known human TFs are downloaded from AnimalTFDB3.0. And the visualization of the core TF regulatory network was performed by Cytoscape software (Shannon et al., 2003). Moreover, the top 10 hub TFs in each module significantly related to the corresponding stage were performed Gene Ontology annotation using the ClusterProfiler package (Ashburner et al., 2000; Yu et al., 2012).

### Associations between transposable elements and transcription factors

The associations between TEs and TFs in human is downloaded from RTFAdb (Karakülah, 2018), which is a public repository of the overrepresented retrotransposon species including LTR retrotransposons, LINEs, and SINEs in the binding sites of the human and mouse TFs. By using ChIP-seq binding profiles of more than 3,000 transcription factors collected from human and mouse samples, RTFAdb can search for more than 1500 retrotransposons on the binding sites of a total of 596 transcription factors. Here, we selected the data of the human embryonic stem cell and iPS cell that were closest to the early embryo type.

## Data Availability

The datasets presented in this study can be found in online repositories. The names of the repository/repositories and accession number(s) can be found in the article/Supplementary Material.

## Supplementary material

Supplementary Figure S1 Related to Figure 1. Validation of TE expression in human and mouse embryos from public data. **(A)** PCA result of early embryo data by Yan et al. (above), and the number of each transposon family in stage-specific transposons (below). **(B)** PCA results of early embryo data by Xue et al. (above), and the number of each transposon family in stage-specific transposons(below). **(C)** Fuzzy clustering analysis of TEs expression signals for the five consecutive stages in mouse. The closer the line color is to red, it means that these are the core TE in the cluster (left). Number of core TE of each class in each cluster. Select transposons with membership value greater than 0.7 as core TE in each cluster and count the number of four (membership>=0.7) (right)

Supplementary Figure S2 Related to Figure 2. **(A)** The number of upregulated genes at each stage of three types of embryos. **(B)** The number of transposons up-regulated from 2- to 4-cell stage in BI embryos overlapped with the transposons down-regulated in uniparental embryos than in BI embryos at the 4-cell stage.

Supplementary Figure S3 The four modules most relevant to the 2-cell to the morula stage. **(A)** Scatterplot of gene significance (GS) for stages versus module membership (MM) in the four modules. GS and MM exhibit a very significant correlation, implying that hub genes of the four modules also tend to be highly correlated with the corresponding stage. **(B)** The expressions of eigengene in the four modules in all samples. The eigengenes of the four modules are all highly expressed in the corresponding stages.

Supplementary Figure S4 The network diagrams of the four modules relevant to the 2-cell to the morula stage. The dot plot shows the comparison of the expression of the top ten TFs among the four modules in the three types of embryos (a, b, c, and d for 2-, 4-, 8-, and morula, respectively). The color of the dot represents the type of embryo, and the shape represents the stage.

Supplementary Figure S5 GO function enrichment analysis of genes in the four modules. Genes in pink module are related to histone modification and methylation, genes in purple modules are related to regulation of gliogenesis differentiation, genes in yellow modules are related to RNA splicing and chromatin assembly, genes in turquoise modules are related to protein transport in the endoplasmic reticulum.

Supplementary Figure S6 The expression of ZSCAN5A target genes in the three types of embryos at the 4-cell stage. The target genes here refer to the related gene of ZSCAN5A in the co-expression network. Significance was calculated using the t test.
