# Supplementary figures and images for: The single-cell expression profile of transposable elements and transcription factors in human early biparental and uniparental embryonic development
Source: Front Cell Dev Biol. 2022 Nov 11;10:1020490. doi: 10.3389/fcell.2022.1020490 (PMC9691860; doi:10.3389/fcell.2022.1020490)

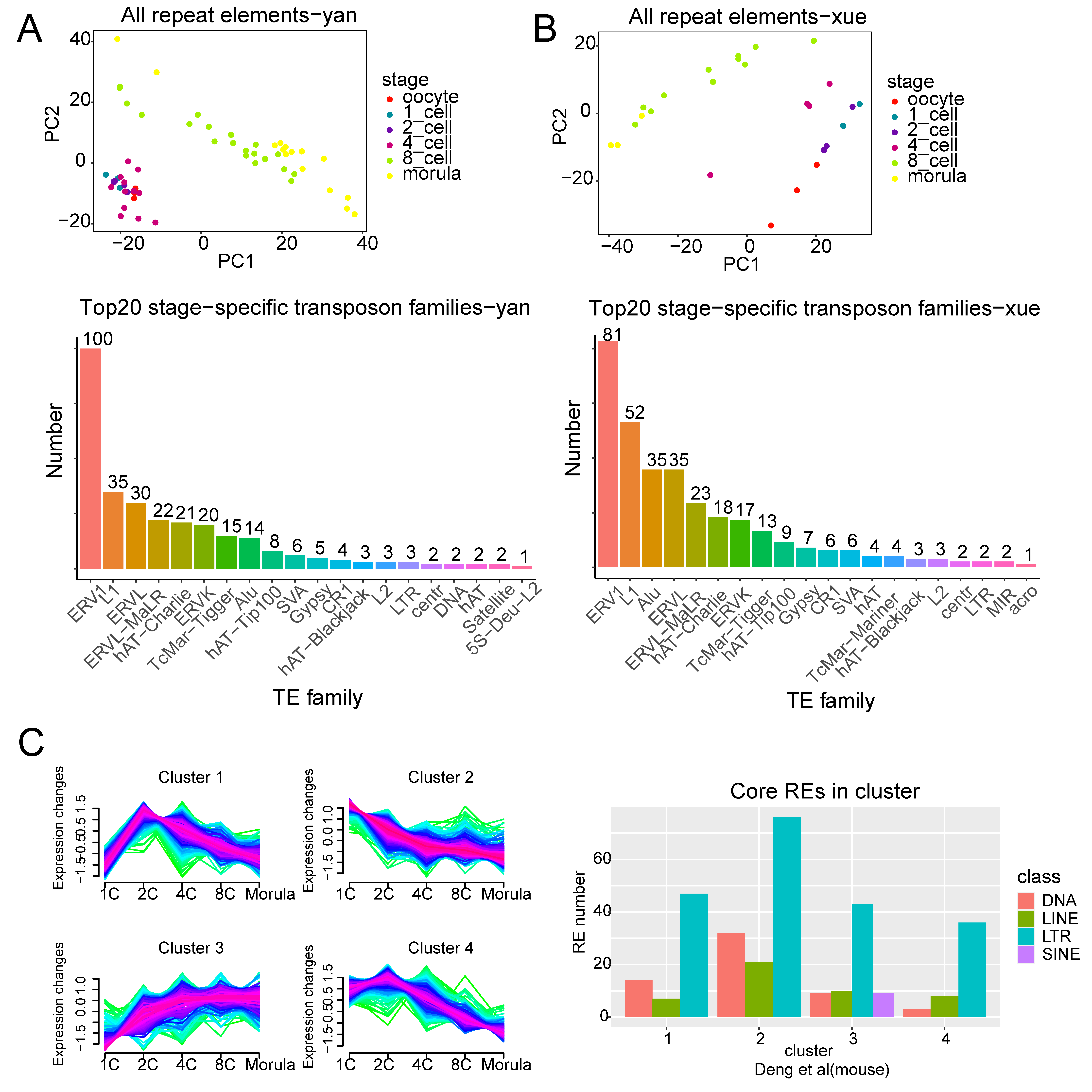

Supplement: Supplementary file 1 [file Image1.tiff]

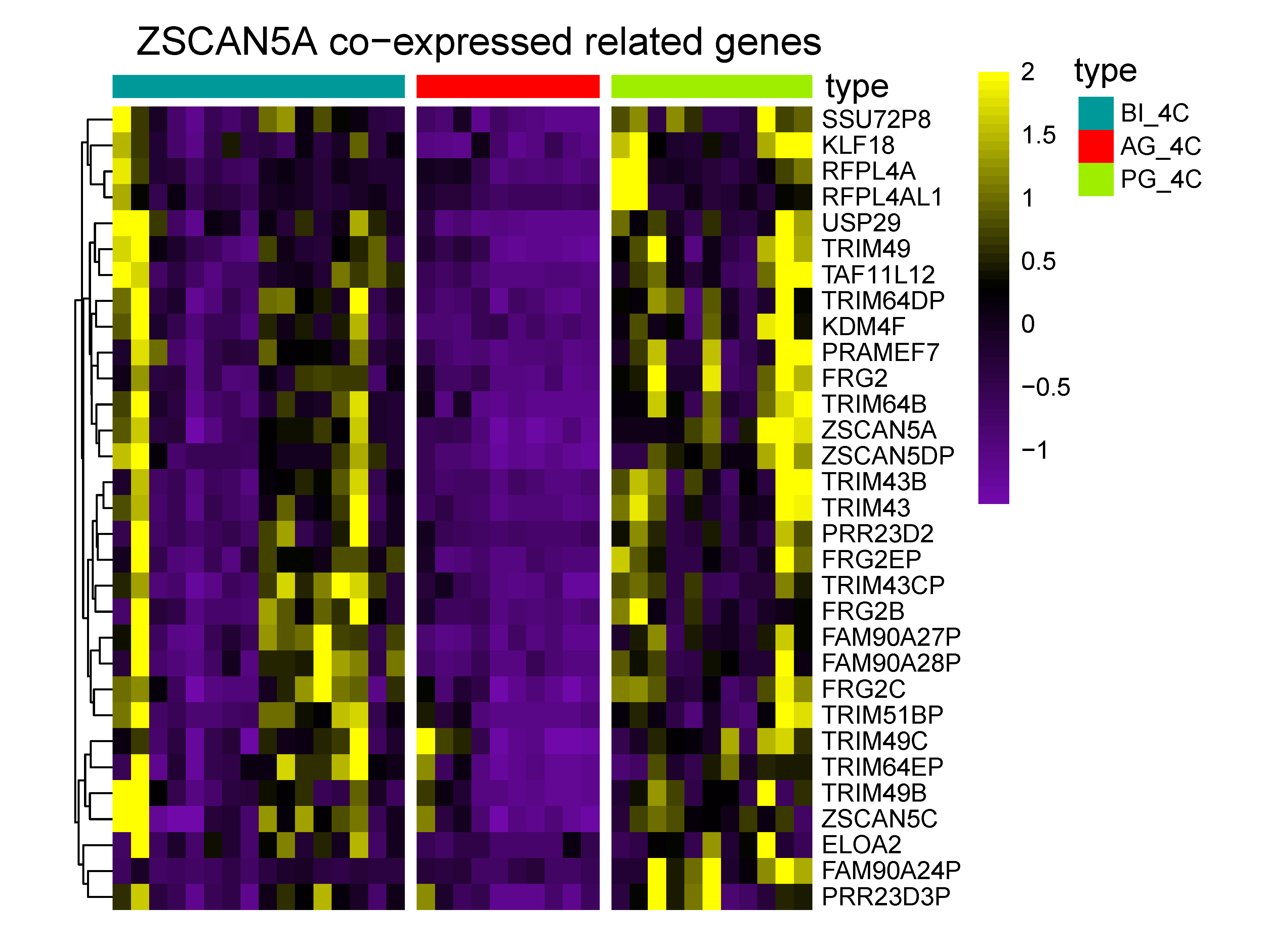

Supplement: Supplementary file 4 [file Image6.tif]

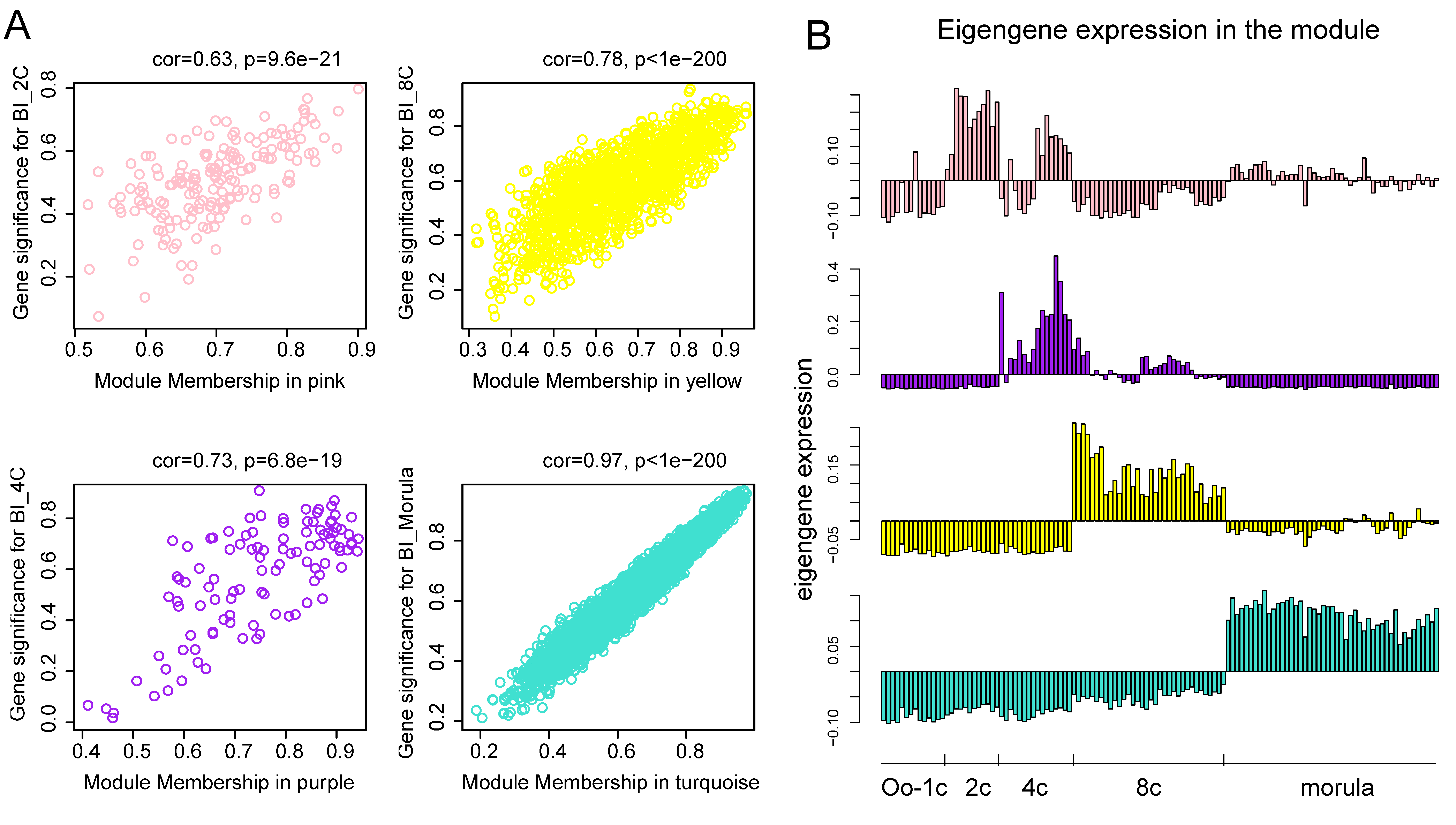

Supplement: Supplementary file 5 [file Image3.tif]

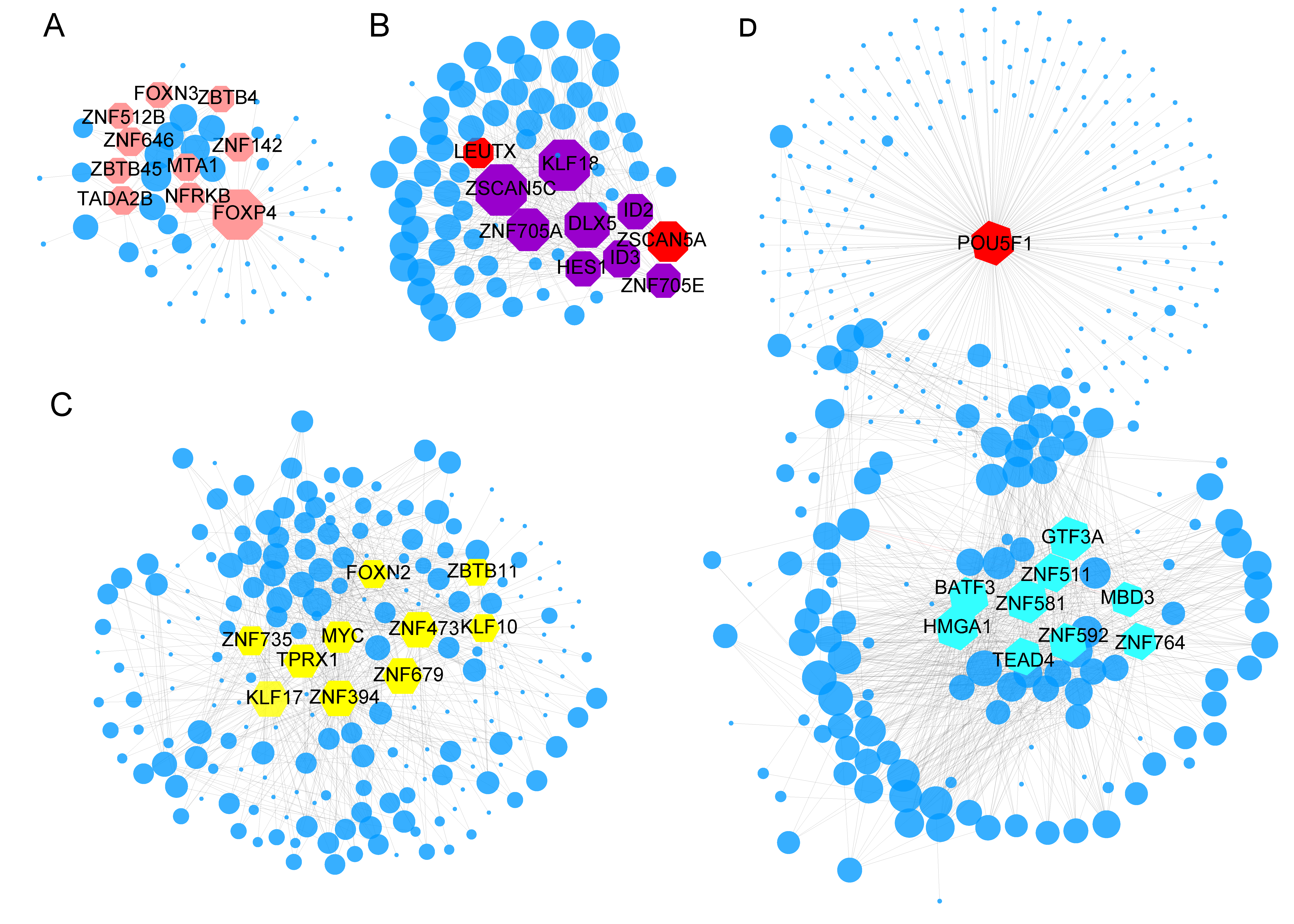

Supplement: Supplementary file 6 [file Image4.tif]

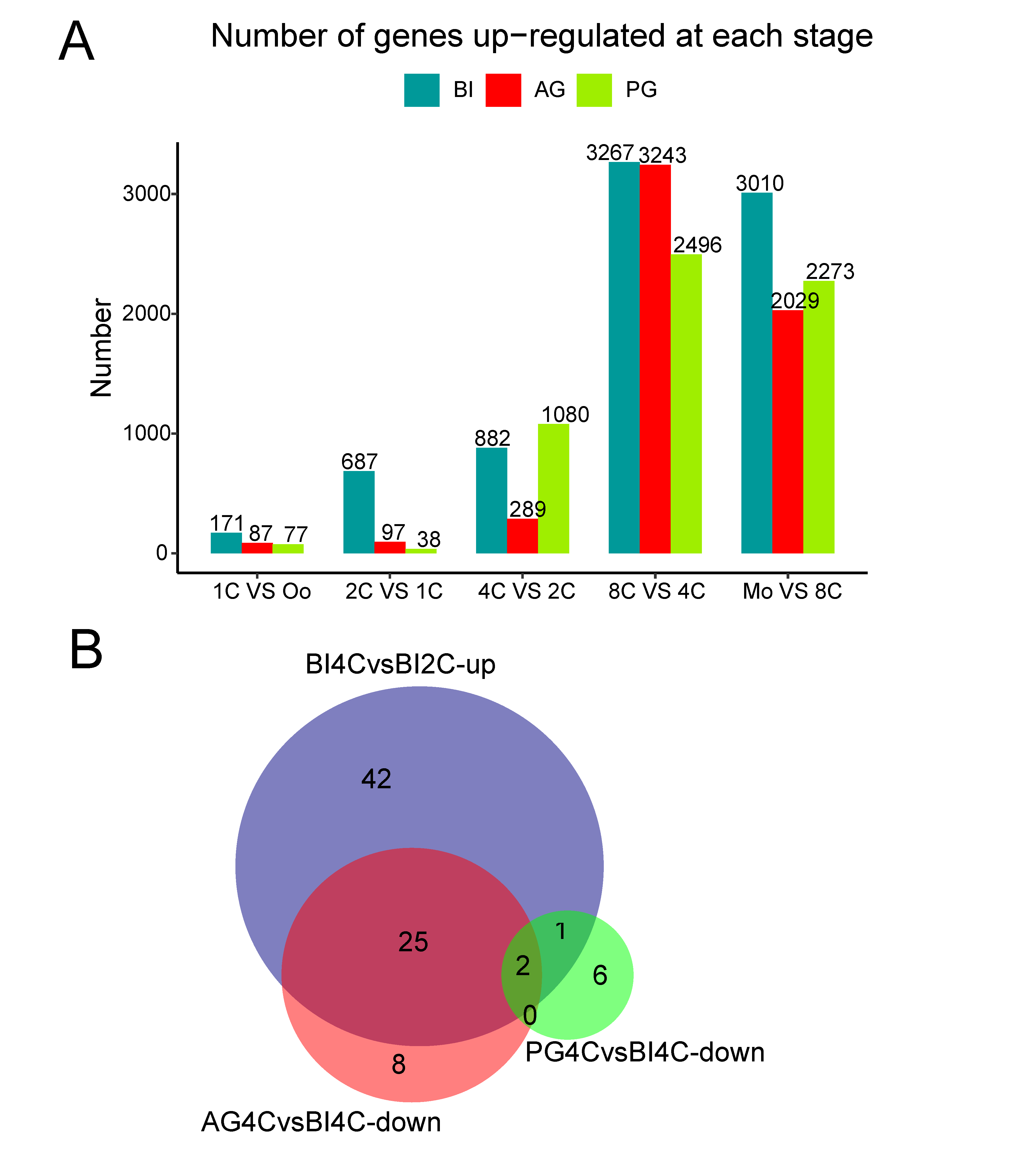

Supplement: Supplementary file 7 [file Image2.tif]
